# Adult-onset medulloblastoma presenting as slow-growing, atypical mass: a case report

**DOI:** 10.1259/bjrcr.20160115

**Published:** 2017-01-25

**Authors:** Anja Gwendolyn van der Kolk, Roeland B van Leeuwen, Louise Poulsen

**Affiliations:** ^1^Department of Radiology, University Medical Center, Utrecht, Netherlands; ^2^Department of Radiology, Gelre Ziekenhuizen, Apeldoorn, Netherlands; ^3^Department of Neurology, Gelre Ziekenhuizen, Apeldoorn, Netherlands

## Abstract

Medulloblastoma accounts for < 1% of all primary central nervous system tumours in adults. Although a “classical” imaging presentation —being a well-defined mass, often located in the cerebellar hemisphere, with surrounding oedema, showing *T*_1_ iso- and *T*_2_ heterogeneous signal intensity and contrast enhancement —has been described, case reports and series have also shown the extremely heterogeneous imaging aspect of this tumour , reflecting its heterogeneous molecular phenotype. Owing to the general location of the tumour within the cerebellopontine angle, causing (fast) progressive symptoms of headache and gait instability, most patients present within 3  months from symptom onset. This case report describes a presentation of adult medulloblastoma not shown before, with an indolent course over a period of 4.5  years, initially without clear abnormalities on imaging. It highlights the importance of including medulloblastoma in the differential diagnosis of all lesions found near/continuous with the fourth ventricle in the adult population, even when clinical onset and imaging characteristics do not suggest “classical” medulloblastoma.

## Case presentation

**Figure 1.**
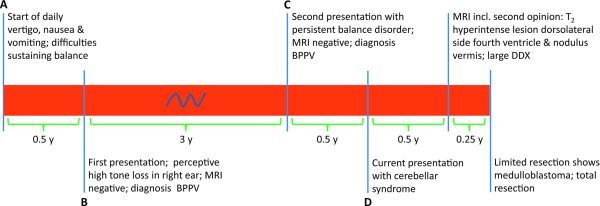
Timeline of clinical symptoms from first onset (left) until total resection of the tumour (right). Specifics for time points A–D can be found in the text. y, years; MRI, magnetic resonance imaging; BPPV, benign paroxysmal positional vertigo; DDX, differential diagnosis.

A 31-year-old male patient first presented to our clinic (time point **A**, Figure 1) with daily vertigo, aggravated by sudden movements of the head and complicated by nausea and vomiting, as well as difficulties sustaining his balance in confined spaces. His medical history was blank except for meningitis at 2 years of age; his family history showed no vertigo, epilepsy or hearing loss and no familial cancer syndromes (such as Gorlin-, Li-Fraumeni-, Turcot-, Gardner- or Cowden syndrome). He had not experienced headaches, and neurologic and audiometric examination only revealed perceptive high tone loss in his right ear with unknown cause; a subsequent MRI examination focused on the cerebellum and cerebellopontine angle half a year later (time point **B**) was normal. A diagnosis of probable benign paroxysmal positional vertigo (BPPV) was made, and the patient was given home exercises to alleviate his symptoms (Figure 1).

Three years later (time point **C**), the patient sought medical attention again because of a persistent balance disorder (his initial symptoms of vertigo had disappeared), present throughout the day with no predilection to one side. Concomitant with these symptoms, he had noticed decreased vision in his lateral visual fields for fast-approaching objects, such as tennis balls. Neurological examination showed a new bilateral horizontal nystagmus when looking laterally, the tandem gait was performed below normal for his age, he had difficulties standing still with closed eyes, and the Dix-Hallpike test (classical test for diagnosing BPPV clinically) was positive with a predilection to the right. Audiometric evaluation showed persistent perceptive high tone loss in his right ear. However, a follow-up MRI examination, including thin slices through the cerebellum and cerebellopontine angle and series after contrast administration, again showed no pathology. The initial diagnosis of BPPV was maintained, and the patient was again given home exercises for symptom alleviation. One-half year later, the patient presented for the third time (time point** D**), and was seen in our dizziness clinic with a balance disorder, difficulties in processing information and in typing (wrong characters), which had been present since 3–4 months after the second MRI examination. Symptoms progressed to dysarthria, decreased vision with diplopia and optokinetic photophobia within the next few months. A full overview of the timeline can be found in Figure 1.

## Clinical findings

Neurologic examination (time point **D**) showed a broad-based, ataxic tandem gait, right-sided eye hypometria, a spontaneous nystagmus that was mostly torsional with extorsion in the left eye and increasing when looking downward and to the right, horizontal saccades and dysdiadochokinesis on the right side. No hypotonia or dysmetria was found; also, there were no signs of elevated intracranial pressure (headache, vomiting). Calorimetric measurements showed bilateral hyperreactivity and a negative Dix-Hallpike test; cerebrospinal fluid analysis and blood workup (including Borrelia test) showed no abnormalities.

## Imaging findings

**Figure 2.**
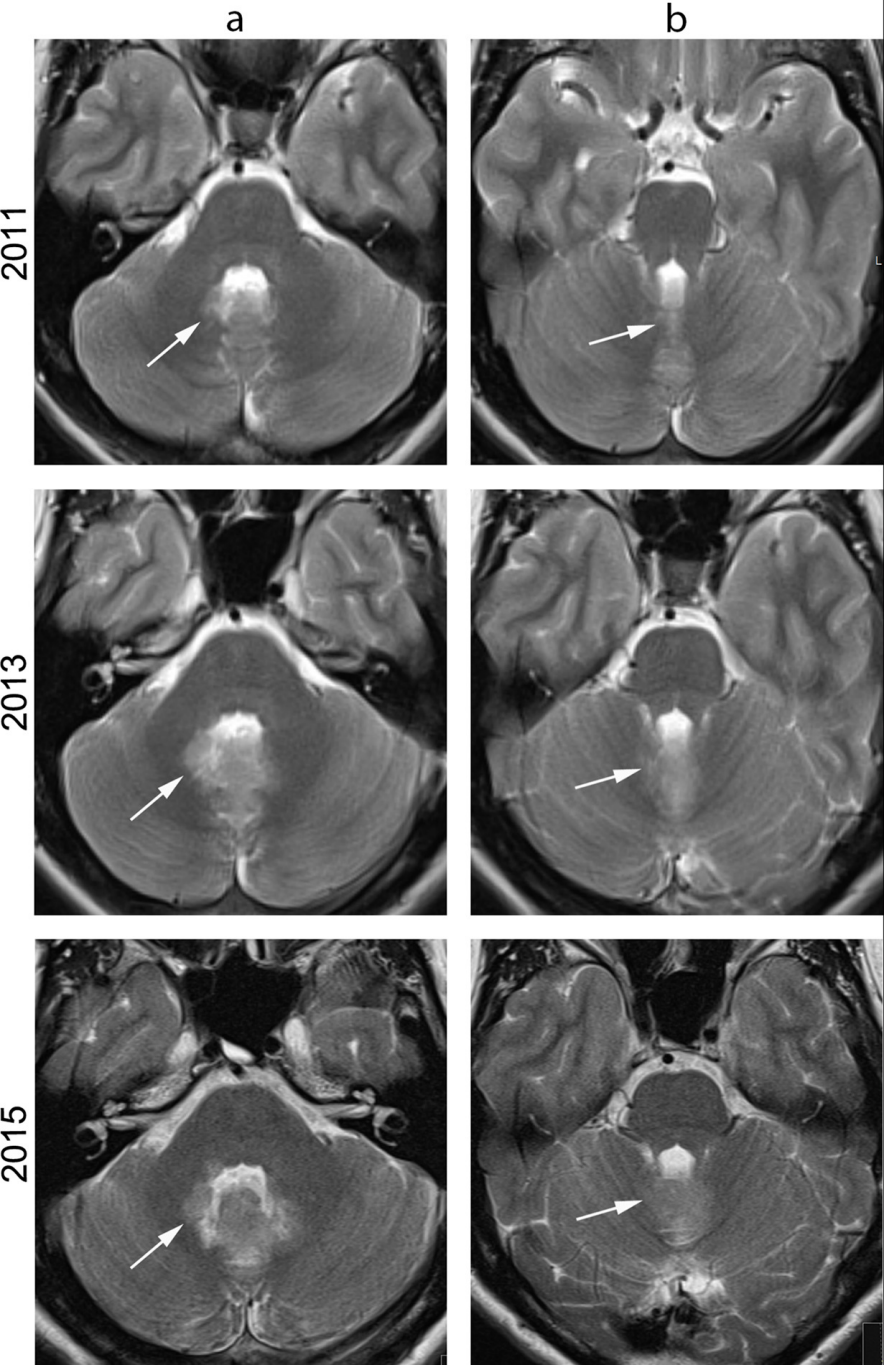
*T*_2_ weighted MR images at the level of the pons (a) and at the level of the mesencephalon (b) at first presentation in 2011 (first row), at second presentation in 2013 (second row) and at time of diagnosis in 2015 (third row). In 2015, an abnormal *T*_2_ hyperintense signal is seen on the dorsolateral side of the fourth ventricle (arrows), with little mass effect. Retrospectively, this area could also be seen on previous MRI examinations, albeit less clearly.

**Figure 3.**
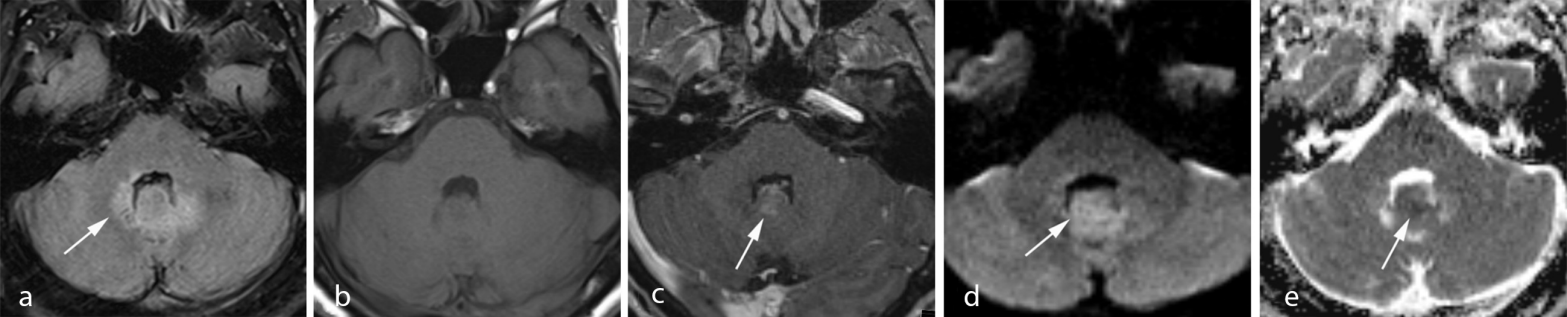
MR images at the time of diagnosis. *T*_2_ fluid-attenuated inversion recovery image on which the abnormal *T*_2_ hyperintense signal on the dorsolateral side of the fourth ventricle and hyperintense nodulus of the vermis can be appreciated (a, arrow). Being isointense to surrounding white matter on *T*_1_ weighted images (b) the area showed subtle, nodular-like contrast enhancement (arrows in *T*_1_ weighted postcontrast image c). Diffusion-weighted imaging showed subtle diffusion restriction (arrows in d (diffusion-weighted image) and e (apparent diffusion coefficient map)).

MRI examination was repeated with a working diagnosis of cerebellar dysfunction. It showed an abnormal, diffuse *T*_2_ hyperintense signal on the dorsolateral side of the fourth ventricle, continuing into the vermis on both sides, without apparent intraventricular mass (Figure 2); the area showed subtle diffusion restriction and subtle nodular contrast enhancement (Figure 3). Compared to previous MRI examinations, on which the areas are visible retrospectively, there was progression without clear mass effect (Figure 2). MR spectroscopy was not performed; an MRI examination of the spinal cord showed no other lesions, and CT examination of the thorax and abdomen showed no primary tumour.

## Differential diagnosis

A slow-growing, diffuse *T*_2_ hyperintense lesion on the dorsolateral side of the fourth ventricle and expanding into the vermis, without intraventricular mass or clear mass effect and with subtle enhancement and diffusion restriction, represents quite a non-specific finding. In children, the differential diagnosis would encompass medulloblastoma, atypical teratoid or rhabdoid tumour, pilocytic astrocytoma, ependymoma and choroid plexus papilloma. The differential diagnosis for this adult also encompassed a variety of pathologies. A low-grade astrocytoma and demyelination can have the same signal intensity characteristics and lack of mass effect, however atypical the location may be. A high-grade tumour, such as a medulloblastoma, ependymoma or metastasis, was found to be less likely because of the slow growth of the lesion and—in case of metastasis—no known primary tumour. The lack of a primary tumour and lack of atrophy also excluded a diagnosis of paraneoplastic cerebellar degeneration. Based on the location a haemangioblastoma or pilocytic astrocytoma was considered, but the characteristic cysts often associated with these tumours were not seen; this was also the case for a Rosette-forming glioneural tumour. L’hermitte Duclos disease was considered; however, this is normally seen in the cerebellar hemispheres and has a characteristic appearance of thickened cerebellar folia, which was not the case here. Neurosarcoidosis and tuberculous leptomeningitis may present with a basal leptomeningitis, sometimes centred around the fourth ventricle with increased signal on *T*_2_ weighted images; however, no other signs of sarcoidosis or tuberculosis were found. Finally, Langerhans cell histiocytosis was considered; however, this presents with more extensive *T*_2_ hyperintense areas in pons, basal ganglia and cerebellar white matter, with mass lesions, which were not seen in our case.

## Treatment

Because of fast clinical deterioration and an extensive differential diagnosis with no clear working diagnosis, the patient underwent a limited resection of the abnormal tissue. Histological examination of the resected tissue showed a medulloblastoma (WHO grade 4). After this resection, clinical symptoms became worse necessitating fast and complete resection of the remaining tumour tissue, with a macroscopically complete resection; however, the tumour borders were not always clearly visible during the procedure.

## Follow-up and outcome

Histopathological examination of the tumour tissue showed a small round cell tumour of the midline, with an immunophenotype consistent with classic type medulloblastoma, with abnormalities in the wingless (WNT) signalling pathway. Postoperatively, dysarthria and cerebellar symptoms—with a predilection to the right side—persisted, and the patient received 36 Gy of craniospinal irradiation with an additional irradiation boost of maximum 55 Gy on the posterior fossa. After 8 subsequent months of physiotherapy, the patient can currently walk independently again, while dysarthria and a light balance disorder remain. Follow-up MRI examinations so far have shown no recurrent tumour.

## Discussion

Medulloblastoma, a malignant brain tumour arising from primitive neuroectodermal cells, is one of the most common primary central nervous system tumours in children; however, it accounts for only < 1% of all primary central nervous system tumours in the adult population.^1,2^ Until recently, it was seen as a single disease that could present both in adults and children. However, recent molecular studies have shown it to comprise of four clinically and molecularly diverse subgroups: WNT, sonic hedgehog (SHH), Group 3 and Group 4.^3^ These four different molecular subgroups have been shown to be unequally distributed between adults and children, they influence age of onset, location and imaging characteristics and are associated with different prognoses.^4^ For instance, the SHH subgroup of medulloblastoma is the most frequent subgroup in adults (62%), these tumours are seen in patients between 20–40 years of age and have an intermediate prognosis, while WNT and Group 4 medulloblastoma are much less common (4–10% and 28% respectively), and are found in a larger age range (18–55 years). WNT has a prognosis of approximately 80%, while Group 4 medulloblastomas have an intermediate prognosis with an overall survival of 75%. Group 3 medulloblastomas are very rare in adults, and have the worst prognosis.^4–6^

The classical clinical presentation of medulloblastoma in both children and adults is that of headache (61%), with or without vomiting, and gait instability (40%). These symptoms are a result of the typical location of medulloblastoma in the cerebellum or surrounding the fourth ventricle. Owing to the disabling nature of these symptoms, patients generally present themselves approximately 12 weeks after symptoms have commenced.^1,2^

In children, medulloblastoma often has the more or less characteristic radiological appearance of a well-defined homogeneous tumour, mainly located within the vermis of the cerebellum, with intense enhancement after contrast administration. On MRI, the tumour is generally *T*_1_ hypointense and *T*_2_ hyperintense. For medulloblastoma in adults, a “classical” imaging presentation has also been described in the literature, consisting of a hypoattenuating, well-defined cerebellar mass, often located in the cerebellar hemisphere, with surrounding vasogenic oedema and obstructive hydrocephalus on CT, while MRI shows a well-defined, *T*_1_ isointense mass with heterogeneous signal intensity on *T*_2_ weighted images, and enhancement after contrast administration.^2^ However, several case reports and case series in the last 20 years have shown the extremely heterogeneous imaging aspect of medulloblastoma in the adult population.^7–9^ In light of the recent discovery of molecular subgroups, this heterogeneity can now actually be seen as a normal reflection of significantly different tumour types, and may aid in the imaging diagnosis of medulloblastoma subtypes.^10^ Our case report adds important features regarding these heterogeneous imaging aspects.

Koci et al^7^ in 1993 reviewed imaging results from 15 adult medulloblastoma patients, and found a mostly classical appearance of the tumour; in a minority of patients, cystic changes and only subtle enhancement were found.^7^ Becker et al^8^ in 1995 found cystic changes to be present in 31% of 13 reviewed cases; all tumours were “classically” iso- to hypointense on *T*_1_ weighted images, heterogeneous hyperintense on *T*_2_ weighted images and 15% showed no enhancement after contrast administration.^8^ Malheiros et al^9^ in 2003 showed similar results in 9 cases of adult medulloblastoma, with isointense signal intensity on *T*_2_ weighted images and cystic/necrotic changes in all tumours as additional findings. Only 1 of 9 patients did not show contrast enhancement.^9^ Our case showed a *T*_1_ isointense tumour, similar to previous case series; however, the tumour was hyperintense on *T*_2_ weighted images and showed no enhancement for the first 4.5 years after symptom onset. Also, no cystic or necrotic changes were present. In all previous cases, molecular subgroup analysis was not yet known. In our case, abnormalities in the WNT signalling pathway were found, suggesting that this tumour belongs to the WNT molecular subgroup. While SHH tumours have been shown to be laterally localized, WNT tumours are located more paramedially, which is concordant with our findings.^10^ Other imaging findings have so far not been correlated with molecular subgroups, probably owing to the low prevalence of medulloblastoma in the adult population.

Although the combination of MRI signal intensities and (lack of) contrast enhancement of our case is relatively unique, the individual characteristics have been more or less described in previous case series. What makes our case interesting is that these imaging features slowly developed over a period of 4.5 years. In most published literature on adult medulloblastoma, time between first presentation and (imaging) diagnosis ranges between 2 months and 1 year.^2,11^ Only one other case report of three cases of adult medulloblastoma reports a time from onset to diagnosis of 1 and 4 years in two of these cases.^12^ However, in the 4-year-case, imaging findings were more severe and an initial diagnosis of L’hermitte Duclos disease was made based on thickened folia of the cerebellum. Also, no MRI examination had been performed in the 4 years before presentation, so that the natural course of the disease could not be illustrated.^12^

When looking at the clinical course of our case, the initial vertigo disappeared (replaced by gait instabilities), while neurologic examination persistently showed perceptive hearing loss of the right ear. Two other cases in the literature have shown hearing loss as the main presenting feature in adult medulloblastoma; however, at least one of these cases showed the tumour mass to be located in the cerebellopontine angle instead of the cerebellar region.^7,13^ In our case the tumour was located on the dorsolateral side of the fourth ventricle, making it less likely that the patient’s perceptive hearing loss can be accounted for by the tumour.

Treatment and prognosis are currently still based on the original view of a single disease. All patients are treated with the most radical surgical resection possible. After surgery, the type of treatment for both adults and children depends on (1) the histological cell subtype (classic, desmoplastic, anaplastic, large-cell or extensive nodularity); (2) the Chang staging system (tumour size and presence of metastases); and (3) the presence of residual disease after surgery.^4^ Cases of medulloblastoma with neither metastases nor residual tumour, and a favorable histology (classic, desmoplastic or extensive nodularity) are seen as average risk and receive postoperative craniospinal radiotherapy. Five-year progression-free survival in adult cases is > 75% with this therapeutic regime. In cases of high-risk medulloblastoma, children receive additional chemotherapy.^4^ In adults, this regime is controversial because it is based on evidence from paediatric population studies; adult studies on the effectiveness of additional chemotherapy are scarce and show conflicting results, and chemotherapy in these patients has been associated with greater morbidity.^14–16^ Although these therapeutic regimes are still used in clinical practice, the recognition of multiple molecular subgroups with different molecular pathways of tumorigenesis has opened up new possibilities for both (dedicated) therapeutics and molecular subgroup-based treatment plans.^4^

## Learning points

1. The presented case illustrates the heterogeneous presentation of medulloblastoma in the adult population, reflecting its heterogeneous molecular phenotype.
2. Adult medulloblastoma does not have a clear “classical” imaging presentation; rather, it ranges from hypo- to hyperintense on *T*_1_ and *T*_2_ weighted images, often showing cystic or necrotic changes, and variably enhances.
3. Medulloblastoma may present with a slow-onset course, instead of the usual subacute presentation within 3 months from symptom onset with a mass in the cerebellum or fourth ventricle.

## Consent

Written informed consent was obtained from the patient for publication of this case report, including accompanying images.
